# Cold-evoked potentials in Fabry disease and polyneuropathy

**DOI:** 10.3389/fpain.2024.1352711

**Published:** 2024-05-15

**Authors:** Dilara Kersebaum, Manon Sendel, Josephine Lassen, Sophie-Charlotte Fabig, Julia Forstenpointner, Maren Reimer, Sima Canaan-Kühl, Jens Gaedeke, Stefanie Rehm, Janne Gierthmühlen, Ralf Baron, Philipp Hüllemann

**Affiliations:** ^1^ Division of Neurological Pain Research and Therapy, Department of Neurology, University Hospital Schleswig-Holstein, Kiel, Germany; ^2^ Schön Clinic Rendsburg, Department of Psychiatry, Psychotherapy and Psychosomatics, Rendsburg, Germany; ^3^ Division of Nephrology, Department of Medicine, Charité, Berlin, Germany; ^4^ Interdisciplinary Pain and Palliative Care Division, Department of Anesthesiology and Intensive Care Medicine, University Hospital Schleswig-Holstein, Campus Kiel, Germany

**Keywords:** neuropathic pain, Fabry disease, polyneuropathy, cold-evoked potentials, diagnostic workup, neurophysiology

## Abstract

**Background:**

Fabry disease (FD) causes cold-evoked pain and impaired cold perception through small fiber damage, which also occurs in polyneuropathies (PNP) of other origins. The integrity of thinly myelinated fibers and the spinothalamic tract is assessable by cold-evoked potentials (CEPs). In this study, we aimed to assess the clinical value of CEP by investigating its associations with pain, autonomic measures, sensory loss, and neuropathic signs.

**Methods:**

CEPs were examined at the hand and foot dorsum of patients with FD (*n* = 16) and PNP (*n* = 21) and healthy controls (*n* = 23). Sensory phenotyping was performed using quantitative sensory testing (QST). The painDETECT questionnaire (PDQ), FabryScan, and measures for the autonomic nervous system were applied. Group comparisons and correlation analyses were performed.

**Results:**

CEPs of 87.5% of the FD and 85.7% of the PNP patients were eligible for statistical analysis. In all patients combined, CEP data correlated significantly with cold detection loss, PDQ items, pain, and autonomic measures. Abnormal CEP latency in FD patients was associated with an abnormal heart frequency variability item (*r* = −0.684; adjusted *p* = 0.04). In PNP patients, CEP latency correlated significantly with PDQ items, and CEP amplitude correlated with autonomic measures (*r* = 0.688, adjusted *p* = 0.008; *r* = 0.619, adjusted *p* = 0.024). Furthermore, mechanical pain thresholds differed significantly between FD (gain range) and PNP patients (loss range) (*p* = 0.01).

**Conclusions:**

Abnormal CEPs were associated with current pain, neuropathic signs and symptoms, and an abnormal function of the autonomic nervous system. The latter has not been mirrored by QST parameters. Therefore, CEPs appear to deliver a wider spectrum of information on the sensory nervous system than QST alone.

## 1 Introduction

Treating neuropathic pain (NP) conditions can be difficult, due to an oftentimes challenging diagnostic workup. Patients with NP present a wide range of sensory signs and symptoms. Various previous studies have employed quantitative sensory testing (QST) to perform sensory phenotyping and subgrouping of the patients to gain new insights into the underlying mechanisms of action and to drive phenotype-based treatment decisions (1–6).

There is an ongoing discussion about whether specific sensory markers are indicative of certain neuropathic syndromes (7). In the case of Fabry disease (FD), the impairment of cold sensation is one of the first evident sensory abnormalities and can serve as an early clinical sign of the disease. FD is an X-linked lysosomal storage disorder characterized by an early disease onset, affecting the nervous system apart from numerous other organ manifestations, such as kidneys, heart, cornea, and endothelial cells (8–11). The damage to organ and nerve tissue originates from an accumulation of toxic metabolites, i.e., globotriaosylceramide and globotriaosylsphingosine [lysoGb3; (12)] due to deficiency of the alpha-galactosidase enzyme, which leads to inflammation, fibrosis, and oxidative stress (13–16). Typically, FD patients report burning pain in the hands and feet (17) triggered by heat or cold stimuli and present abnormal cold detection thresholds in the upper limb (18) and cold sensory loss (19–21), indicative of a small fiber neuropathy (22). Early detection of the disease is of utmost importance since severe neuropathic and cardiovascular complications may be prevented by specific enzyme replacement therapy (9, 23). It has been reported that small fiber dysfunction is more common than large fiber dysfunction in the early stages of FD (24), making the detection of a small fiber dysfunction crucial in the early diagnosis of FD. Over the past decade, there has been growing interest in an electrophysiological method for examining cold-mediating, thinly myelinated Aδ-fibers and their central pathways: the recording of cold-evoked potentials (CEPs) (25–27). Previously, in healthy subjects, both our research group and colleagues from other research laboratories have demonstrated CEPs to be a reliable, non-invasive tool for measuring Aδ-fiber integrity with various thermodes and even stimulation parameters (25, 28–31).

While laser-evoked potentials (LEPs) have long been recognized for their assessment of nociceptive pathways (32) and given the progress with CEPs in healthy individuals (31, 33), the clinical relevance of cold-evoked potential (CEP) recordings—especially regarding diagnosing NP—is promising but subject to ongoing discussion, due to scarcity of patient data available (27, 34). In a previous study, we introduced cases of a patient with central pain and another with polyneuropathy (PNP), both demonstrating abnormal CEPs corresponding to their clinical findings and providing initial promising insights into their potential for clinical and diagnostic application (28). Recently, Perchet et al. reported on CEPs in patients with suspected NP compared to LEPs. They concluded that “for some patients suffering from symptoms limited only to cold, CEPs but not LEPs may allow the diagnosis of thin fiber pathology” (30).

In earlier projects, we conducted comparisons of CEPs between single patients with neuropathic conditions and healthy controls (28, 35) and found possible associations between abnormal CEPs and the occurrence of pain and sensory loss. However, we were unable to draw any definitive conclusions as these preliminary insights were based on single-patient cases. We assumed that abnormal CEPs reflect an abnormal small fiber function potentially due to a significant reduction of intraepidermal nerve fibers reflecting neuropathic damage. A similar reduction in intraepidermal Aδ-fibers was observed, for instance, in patients with chemotherapy-induced painful PNP, a group known to experience NP, especially thermal hyperalgesia (36–38). We proceeded with a CEP patient study in an attempt to validate these assumptions.

This study aims to determine whether CEPs are feasible in FD patients and, if applicable, whether they can detect abnormal function of cold-mediating Aδ-fibers using a CE-certified thermal stimulator for clinical/diagnostic use. Subsequently, we investigated patients with PNP as representatives of a more heterogeneous NP syndrome (7, 39). We focused on whether CEPs correlate with the sensory phenotype and whether abnormal CEP findings are associated with an altered or abnormal sensory phenotype (assessed by QST). We hypothesized that patients with abnormal CEPs would report higher pain levels, be more likely classified as neuropathic based on the painDETECT questionnaire, and, in the case of FD patients, achieve higher scores on the FabryScan.

## 2 Materials and methods

### 2.1 Study cohort and design

Patients with FD and PNP and age-matched healthy subjects were recruited. According to the general inclusion/exclusion criteria (see Supplementary Material), subjects with current alcohol or drug abuse, who are pregnant or breastfeeding women, with insufficient language skills, or who are unable to give informed consent were not eligible for the study. Patients with FD, with and without pain, were included only with a mutation in the GLA gene and with a completed clinical evaluation. Both currently available medical laboratory testing services [ARCHIMEDlife (40) and CENTOGENE (41)] have been used in some of the patients, while others have been diagnosed by genetic panel testing. There was one patient with a stroke history (*n* = 1) who was not excluded from the analysis as organ manifestations of FD (including the endothelium) are broad. PNP patients with and without pain were recruited, and the inclusion criteria were based on the consensus criteria for a symmetric PNP (42). Healthy subjects were, inter alia, only eligible for participation if they met the following criteria: absence of neurological and pain disorders and the non-usage of analgesic medication within the past 14 days. Additionally, patients who were diagnosed with psychosis, depression, anxiety, panic attacks, eating disorders, chronic fatigue or exhaustion, addiction or dependence, or any other severe organ system failures were excluded as recommended by Gierthmühlen et al. (43). FD patients, PNP patients and healthy subjects underwent CEP and QST assessment. Apart from a few exceptions, QST and heart rate (HR) variability have been assessed in all subjects prior to this study (44). In patients, both feet were investigated (the most affected side was included for analysis). For healthy subjects, no side-dependent evoked potential (EP) differences were known (26, 45); therefore, the right body side was chosen for the test application. Both patient groups completed the PDQ. The Fabry group was additionally characterized with the FabryScan, whereas the PNP group underwent nerve conduction studies.

In accordance with the Declaration of Helsinki, patients and controls were first explained the aim and nature of the tests and provided their written informed consent. The study was approved by the Ethics Committee of the University Hospital of Kiel (study protocol number: A 101/15) and registered in the German Clinical Trials Register (DRKS00009013).

### 2.2 Demographic data

Age, sex, comorbidities, medication, and height of patients and healthy volunteers were assessed and are reported in the Results section.

### 2.3 CEPs

The subjects were positioned on a comfortable stretcher. To prevent blinking or eye movement artifacts, they fixed their gaze on a marking on the ceiling and were asked not to blink or to move their eyes during stimulus application and 3 s until a ping tone chimed. The latter signaled the subjects to rate the perceived cold stimuli on a numerical rating scale (0 = not cold at all, 10 = most imaginable cold). The room temperature was maintained at a constant 22°C. The skin temperature of the testing sites was measured before the testing started and was >32°C at the test site (28, 35). There was no standard wait time before starting the recordings. Cold stimuli were applied to the dorsum of the hand and the foot using the PATHWAY Pain & Sensory Evaluation System (Medoc, Israel; CE-number 0473; software version 4.0.11.0). A baseline temperature of 30 °C, a destination temperature of 25°C, a destination rate of 20°C/s, and a return rate of 40°C/s were used. In total, 25 CEPs with an interstimulus interval of 8–12 s were applied on each test site. The thermode remained at a fixed position (28).

Gold cup electroencephalography (EEG) electrodes (Fz, Cz, Pz, C3, C5, C4, C6, T3, and T4) were attached according to the international 10–20 system and referenced to linked earlobes for the recording of potentials. A grounding electrode was attached to the torso. An electrooculogram (EOG) was used for detecting eye movement/blinking artifacts. An artifact correction step based on regression has been applied to account for these artifacts. The EEG was recorded with Brain Vision Recorder 1.2 using the BrainAmp MR plus EEG amplifier (Brain Products GmbH, Gilching, Germany) and analyzed with Brain Vision Analyzer 2.0 (Brain Products GmbH; Gilching, Germany, version 2.0.3.6367). The EEG was band-pass filtered with 0.3–35 Hz, and the sampling rate was 1,000 Hz (25, 35).

Both peak detection and artifact rejection were performed manually and framewise. For each frame, a baseline correction was performed using a pre-stimulus window from −500 to −100 ms to the N1 peak for the determination of the N1. The N2P2 amplitude was measured from the most negative (N2) to the most positive peak (P2). The latency of each component was measured from the stimulus onset (0 ms) to the most negative (N2) and most positive (P2) peaks of the averaged potentials, respectively. The amplitude and latency data were measured at the Cz channel. The other channels were used to support the correct identification of the averaged potential and for artifact detection.

### 2.4 Quality criteria for inclusion into the CEP analysis

All frames containing artifacts or analysis-hindering elements 0.5 s before the stimulus and 2 s afterward due to movement or blinking were excluded from the analysis. Blinking artifacts were identified and taken care of with an ocular correction filter. Disruptive factors such as the occurrence of alpha-EEG sequences or muscular artifacts were excluded during manual inspection. CEP recordings were included in the final analysis if the recording of the subjects contained at least 50% artifact-free EEG segments (i.e., 13/25 EEG frames). On average, 73.5 ± 20.4% of the segments from the foot and 73.6 ± 20.7% from the hand were used for the analysis.

### 2.5 QST

Small fiber function was assessed with QST according to the protocol of the German Research Network on Neuropathic Pain (DFNS) (46–48). QST results were defined as abnormal if the *Z* value was outside the limits of a 95% confidence interval of healthy controls of the DFNS database (46).

Thermal stimuli were applied using a thermal testing device (TSA 2001-II, Medoc, Israel), which applied cold stimuli with a ramp of 1°C/s reaching a minimal temperature of 0°C. Cold detection threshold (CDT), the parameter for cold detection of the QST protocol, was assessed in both feet while the foot with the higher threshold and the ipsilateral hand dorsum were chosen for further testing (49–51).

### 2.6 Questionnaires

#### 2.6.1 PDQ

Originally developed as a screening tool for the detection of a NP component in patients with chronic pain (52), the PDQ by now has also been shown to enable the identification of neuropathic subgroups and sensory profiles (6). This questionnaire assesses general pain intensity, the course and distribution of pain, and finally the following sensory signs: burning sensation, tingling/prickling, painful to light touch, sudden pain attacks, painful to cold or heat, numbness, and pressure pain. These are then rated on a six-point Likert scale (0 = never, 5 = very strongly). The sum of all components results in a score ranging from 0 to 38. A sum score of ≥19 indicates a >90% probability that a NP component is present while ≤12 point makes it unlikely.

We have used painDETECT in its version 2010.

#### 2.6.2 FabryScan

The FabryScan is a screening tool specifically developed for the identification of FD patients (53). It consists of 10 items covering different typical symptoms for FD and differential diagnoses and three bedside exams. With a score of 16 or more, FD is considered likely. A score between 11 and 15 is interpreted to be an unclear result.

We have used the FabryScan in its original validated version.

### 2.7 Autonomic measures

We recorded the HRV by a three-lead electrocardiogram during a 5 min resting period and during controlled breathing for a period of 110 interbeat intervals. We also assessed the root mean square of successive square differences (RMSSD) in a resting and breathing (RMSSDb) condition. A computer-assisted equipment and software (ProSciCard III, MediSyst GmbH, Germany), developed according to the 1996 Task Force Guidelines, was used to process and analyze the HRV (1996). This analysis algorithm detected artifacts and extrasystoles as well as dismissed a series with an artifact percentage of >10% (54). The abovementioned software was also used to conduct a time-domain measurement (TDM) during orthostatic conditions, where out of 50 RR intervals, the respiratory cycle with the highest and lowest HRV is detected and the quotient is calculated (HR max/HR min).

For both the RMSSDb and orthostatic TDM, higher values were rated as favorable as patients with cardiac conduction abnormalities such as atrial fibrillation would have been recognized by the program and excluded from the analysis (neither PNP nor FD patients presented atrial fibrillation).

### 2.8 Statistical analysis

Statistical analysis was conducted using SPSS (SPSS 29.0; SPSS, Inc., Chicago, IL, USA). All parameters were displayed as mean (± standard deviation). CEPs were defined to be abnormal if their N2P2 amplitude was below the lower limit of a 95% confidence interval of age-matched controls' artifact-free average or if the N2 latency was above the upper limit of a 95% confidence interval of age-matched healthy controls.

#### 2.8.1 Group comparisons

For the comparison of group variables (height, N2 and P2 latencies, and N2P2 amplitudes of the hand and foot, the CDT of the hand and foot, and the PDQ and FabryScan score if applicable), the Mann–Whitney *U* (MWU) test was used. Subgroup analyses on painful vs. painless patients or FD patients with or without abnormal CEP parameters were also conducted using the MWU test. FD patients and healthy controls were age-matched during the recruitment phase and therefore did not differ significantly in age (first step of the study). As expected, the PNP cohort (average onset of disease in middle-aged and older patients) was significantly older than the Fabry cohort (average onset of disease in late childhood) and the controls (who were age-matched to FD). The PNP cohort was recruited during the second step of the study. Before performing group comparisons, we therefore performed an age matching procedure, excluding the oldest PNP patients and the youngest healthy controls, so that age would not differ significantly between PNP patients and controls or to FD patients.

#### 2.8.2 Correlation analyses

To detect associations between CEP data, QST items, autonomic items, and questionnaire scores, a correlation analysis was performed using Spearman’s rho. Correlation analyses were performed in all patients and each patient group (PNP, FD) separately. The significance level (*p* < 0.05) was adjusted for multiple testing by multiplying it with the number of analyzed items.

## 3 Results

### 3.1 Group characterization

A total of 16 patients with FD (age 44.25 ± 17.92 years, 11 females, 5 males), 21 patients with PNP (age 64.62 ± 11.19 years, 9 females, 12 males), and 23 controls (age 46.83 ± 19.53 years, 13 females, 10 males) were recruited. Five FD patients were on enzyme replacement therapy (agalsidase, *n* = 3; migalastat, *n* = 2). Although female patients were heterozygous (most common in FD), some received enzyme replacement therapy due to measurable organ damage. Some patients did not fulfill the criteria for treatment at the time of study conduction (e.g., low symptom burden in combination with a “non-classical mutation”). Some patients with eligible mutations received chaperone-therapy instead of enzyme replacement.

The PNP group offered the following etiologies: diabetic (*n* = 5), HMNS type II (*n* = 1), CIDP (*n* = 1), paraneoplastic (*n* = 2), alcohol-related (*n* = 2), and chemotherapy-induced (*n* = 2). Eight of the 21 patients remained of unclear origin. The MWU revealed no significant differences in height between patients with FD and controls/age-matched patients with PNP and controls.

**Figure 1:**
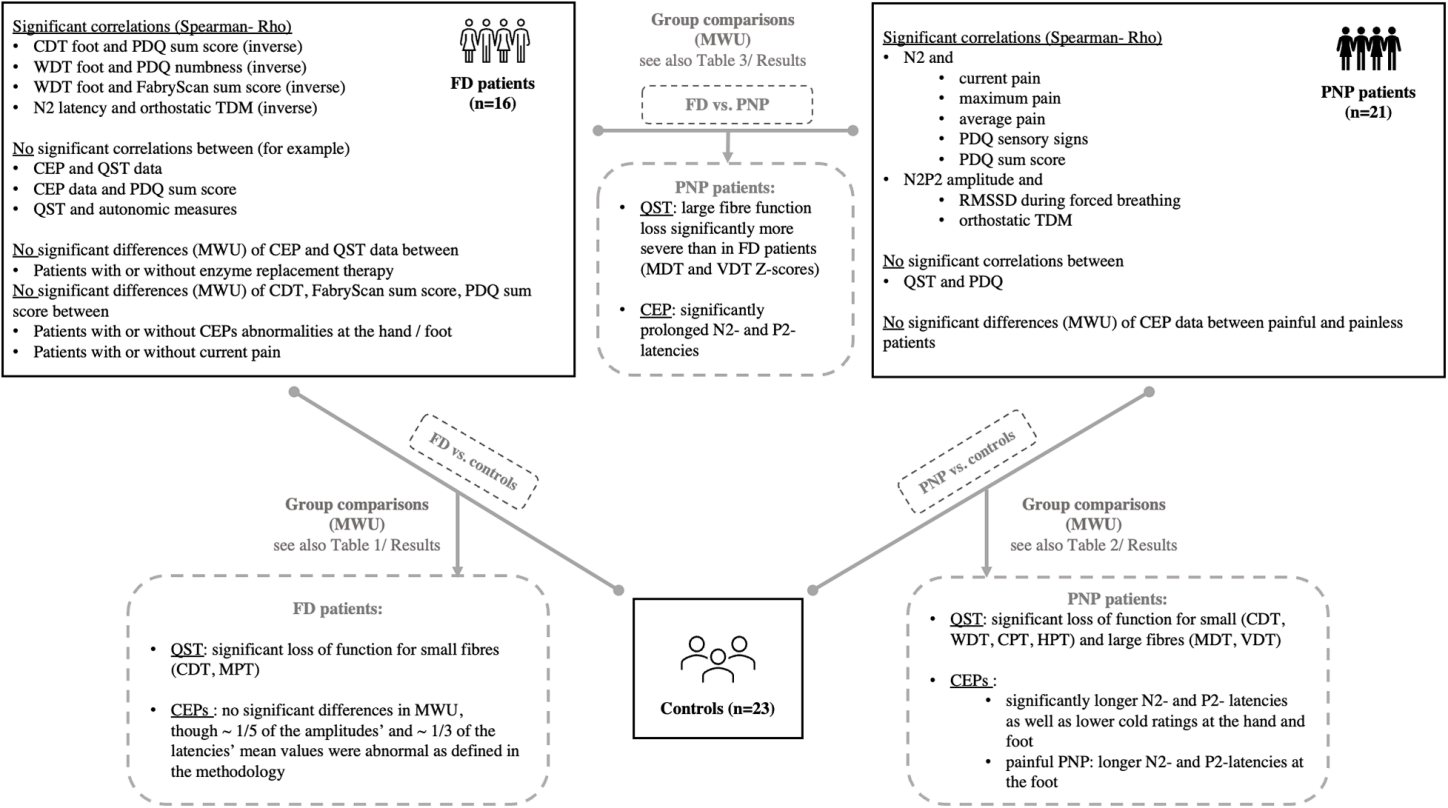
Overview of the study population (controls, PNP and FD patients) and the performed statistical analyses with a summary of significant results after correction for multiple testing. For comprehensive data, see the Results section of the manuscript. CDT, cold detection threshold; CEP, cold-evoked potential; CPT, cold pain threshold; HPT, heat pain threshold; FD, Fabry disease; MDT, mechanical detection threshold; MWU, Mann–Whitney *U*; PDQ, painDETECT questionnaire; PNP, polyneuropathy; TDM, time-domain measurement; QST, quantitative sensory testing; WDT, warm detection threshold; VDT, vibration detection threshold.

**Figure 2:**
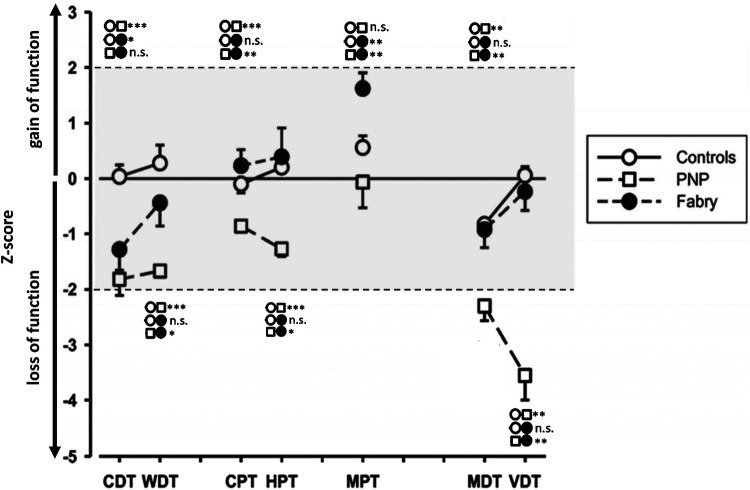
Quantitative sensory testing (QST) profiles of controls and PNP and FD patients. The asterisks indicate significant group differences; *** ≙ *p* < 0.001; ** ≙ *p* < 0.01; * ≙ *p* < 0.05; group symbols are indicated on the right side of the figure. FD patients exhibit a loss of Aδ-function, while the PNP group exhibits a wide range of dysfunction with functional loss of small and large fibers. The controls are within the normative range of *Z*-values. CDT, cold detection threshold; WDT, warm detection threshold; CPT, cold pain threshold; HPT, heat pain threshold; MPT, mechanical pain threshold; MDT, mechanical detection threshold; VDT, vibration detection threshold; FD, Fabry disease; PNP, polyneuropathy.

**Figure 3:**
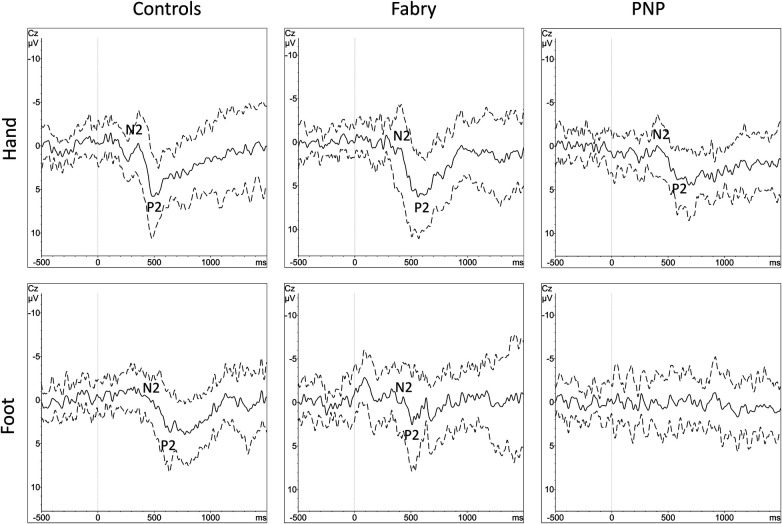
Grand averages of CEPs with standard deviations. Grand averages of CEPs derived from the hand and foot are displayed for each group (controls, FD and PNP patients). The black line equals the averaged EP of each cohort, the dashed line equals one standard deviation of the averaged EP data. N2 and P2 markers indicate the CEP potential. Within the PNP group, at the foot, there was no identifiable grand-average potential due to heterogenous EP latencies caused by heterogenous loss of function in different individuals. CEP, cold-evoked potential; EP, evoked potential; FD, Fabry disease; PNP, polyneuropathy.

**Figure 4:**
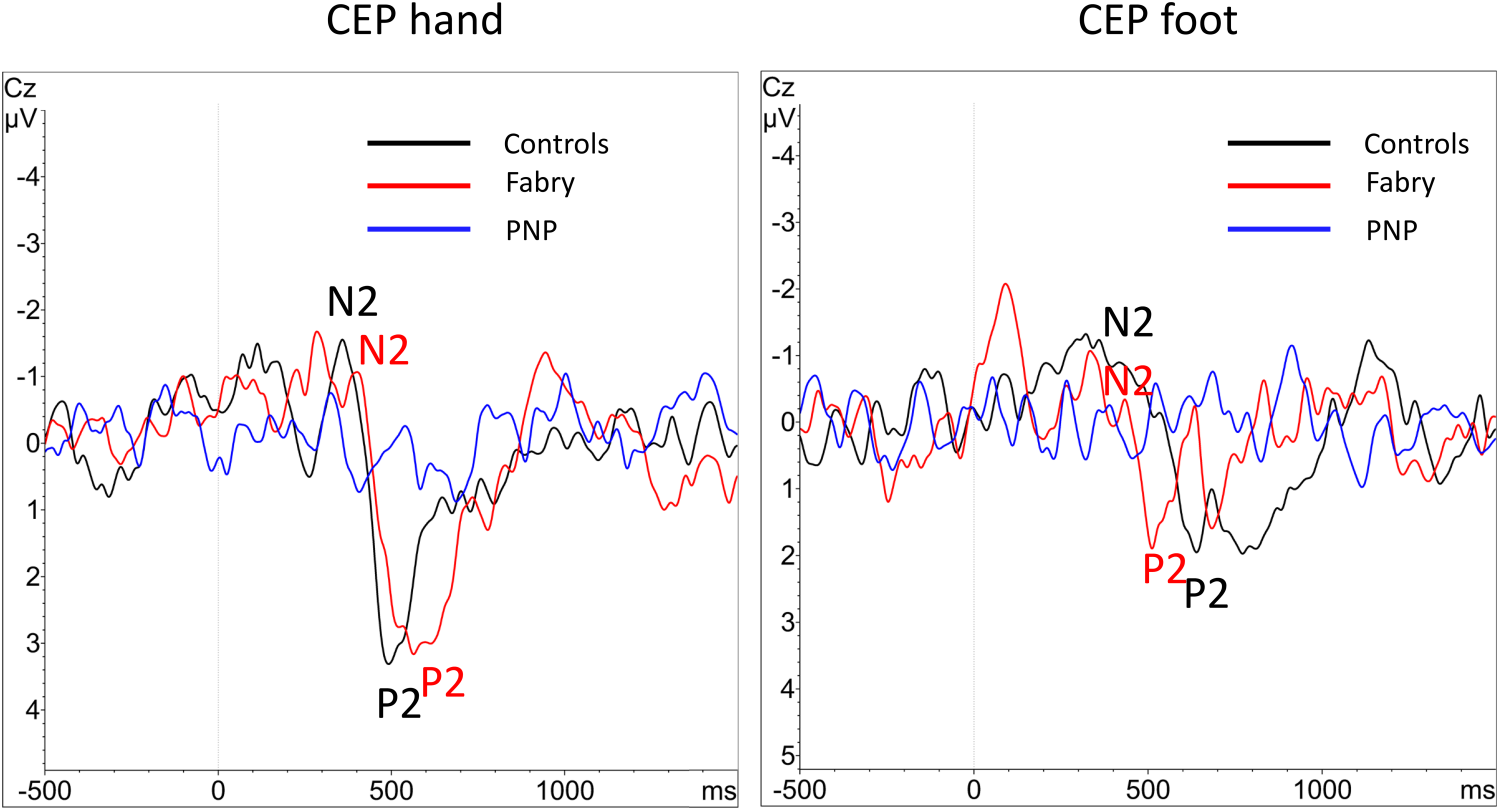
Overlay of grand-averaged CEPs. After performing the grand average of the CEP data there were no visible averaged CEPs within the PNP group at the hand and foot, due to the heterogeneity of normal and abnormal values. N2 and P2 markers indicate the CEP potential where applicable. CEP, cold-evoked potential; EP, evoked potential; PNP, polyneuropathy.

**Figure 5:**
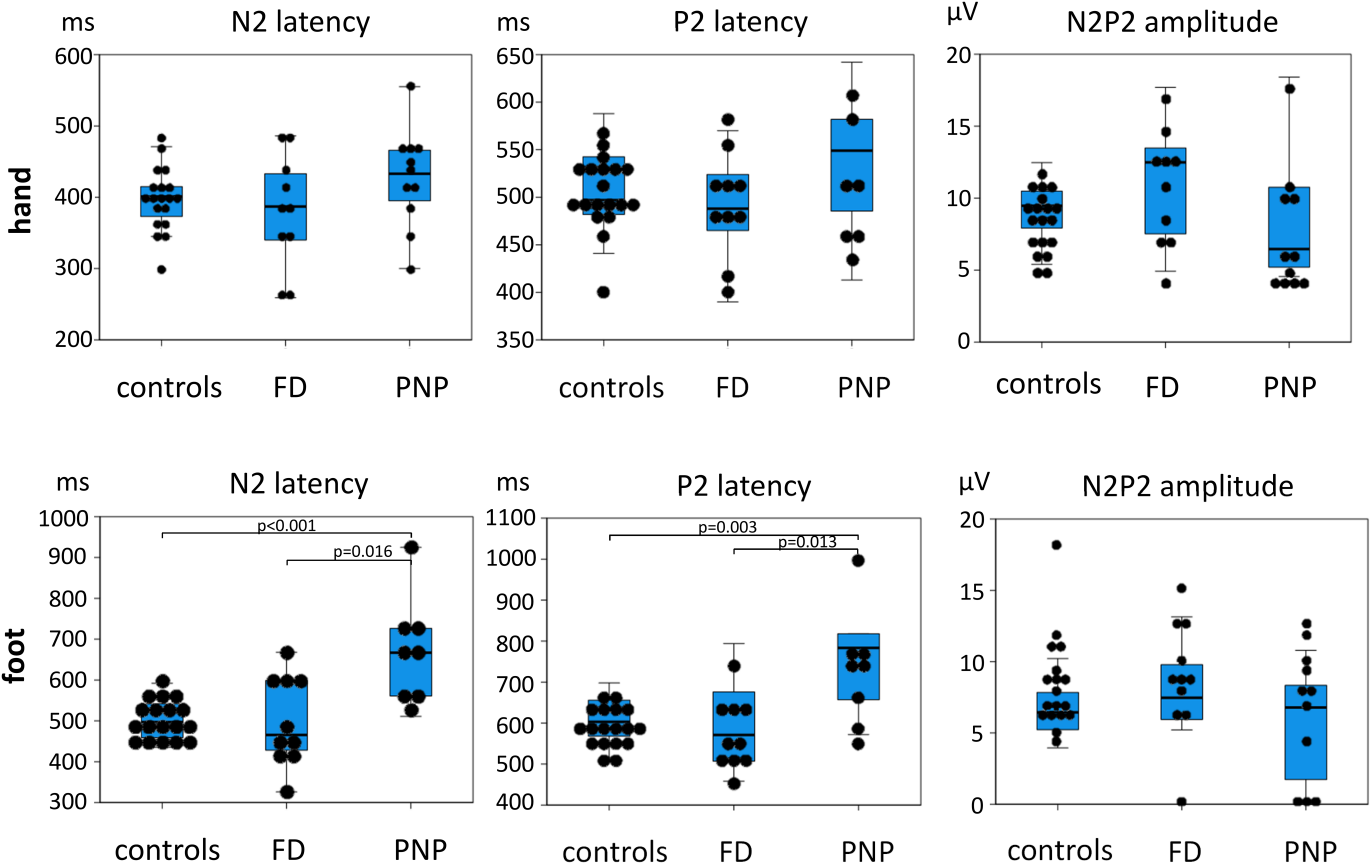
Dot blot and box blot of CEP data. N2 and P2 latencies as well as N2P2 amplitudes of controls and FD and PNP patients are shown here. The dots indicate individual patient data. The box blots indicate minimum and maximum values (T-bars) first quartile, median, and third quartile. There is a strong latency difference between PNP patients and controls, but not between FD patients and controls, supporting that PNP patients exhibit a more severe small fiber dysfunction than FD patients (see also Figure 2). FD, Fabry disease; PNP, polyneuropathy.

Epidemiological data, EP values, and questionnaire scores are presented in Table 1. Since PNP patients had a significantly higher age as compared to FD patients and healthy controls, we performed a secondary age matching between the PNP group and healthy controls as shown in Table 2. Table 3 provides an age-matched comparison of patients with FD and PNP. Supplementary Tables A and B (see Supplementary Material) provide an overview of abnormal results (indicated through “X”). Figure 1 gives an overview of the studied groups and the performed statistical analyses with a brief summary of the significant results. Figure 2 shows the *Z*-score sensory profiles of both the patient groups and the controls. EEG data of the hand and foot for each group respectively is displayed in Figures 3, 4, and 5.

**Table 1:** Demographic data.

	FD		PNP		Control	
	*n*	mean ± SD	*n*	mean ± SD	*n*	mean ± SD
Age (years)	16	44.25 ± 17.92 (34.7; 53.8)	21	64.62 ± 11.19 (59.53; 69.71)	23	46.83 ± 19.53 (38.38; 55.27)
Height (cm)	16	168.69 ± 8.36 (164.23; 173.14)	21	178.57 ± 9.11 (174.43; 182.72)	23	174.13 ± 8.78 (170.33; 177.93)
N2 hand (ms)	13	369.00 ± 76.60 (322.71; 415.29)	15	414.27 ± 76.23 (372.05; 456.48)	23	388.00 ± 45.61 (368.28; 407.72)
P2 hand (ms)	13	473.54 ± 77.21 (426.88; 520.2)	15	519.73 ± 85.81 (472.21; 567.25)	23	496.57 ± 52.65 (473.8; 519.33)
Amplitude hand (µV)	13	11.69 ± 3.67 (9.47; 13.9)	16	7.69 ± 4.5 (5.29; 10.09)	23	10.07 ± 2.80 (8.86; 11.28)
Cold rating hand dorsum	16	2.94 ± 1.95 (1.9; 3.98)	21	2.07 ± 1.47 (1.4; 2.74)	23	3.23 ± 1.37 (2.64; 3.82)
N2 foot (ms)	13	487.54 ± 98.93 (427.76; 547.32)	11	645.73 ± 174.75 (528.33; 763.13)	22	501.36 ± 49.46 (479.43; 523.29)
P2 foot (ms)	13	583.15 ± 95.30 (525.56; 640.74)	11	741.18 ± 188.21 (614.74; 867.63)	22	607.36 ± 50.68 (584.89; 629.83)
Amplitude foot (µV)	14	8.02 ± 3.59 (5.95; 10.1)	18	5.16 ± 5.82 (2.26; 8.05)	22	8.29 ± 3.93 (6.54; 10.03)
Cold rating foot dorsum	16	2.63 ± 2.27 (1.42; 3.84)	21	1.36 ± 1.75 (0.57; 2.16)	23	3.02 ± 1.54 (2.36; 3.69)
CDT foot *Z* value	16	−1.29 ± 1.37 (−2.01; −0.56)	21	−1.84 ± 1.43 (−2.49; −1–19)	23	0.04 ± 0.99 (−3.89; 0.46)
PDQ score	16	15.94 ± 8.505 (11.41; 20.47)	21	13.71 ± 8.84 (9.69; 17.74)	n/a	n/a
FabryScan score	14	16.14 ± 4.33 (13.64; 18.64)	n/a	n/a	n/a	n/a

Cold-evoked potential (CEP) values and questionnaire scores for each subject group.

FD, Fabry disease; PNP, polyneuropathy; PDQ, painDETECT questionnaire; CDT, cold detection threshold. The lower and upper 95% confidence limits are presented in brackets below the mean ± standard deviation. Patients with clearly abolished potentials were given an amplitude of “0,” hence the partial discrepancy of the patient numbers for latencies and amplitudes.

**Table 2:** Demographic data, CEP values, and questionnaire scores for PNP patients and healthy cohort after age matching.

	PNP		Healthy		Significance level (*p*)
	*n*	mean ± SD	*n*	mean ± SD	
Age (years)	13	57.62 ± 7.74 (52.94; 62.29)	19	52.21 ± 17.01 (44.01; 60.41)	ns
Height (cm)	13	180.15 ± 8.66 (174.92; 185.39)	19	174.32 ± 9.27 (169.85; 178.78)	ns
N2 hand (ms)	11	425.82 ± 67.80 (380.27; 471.37)	19	397.42 ± 43.95 (376.24; 418.60)	ns
P2 hand	11	532.73 ± 72.38 (484.10; 581.35)	19	505.74 ± 50.48 (481.41; 530.07)	ns
Amplitude hand (µV)	11	8.24 ± 4.25 (5.39; 11.1)	19	9.16 ± 2.03 (8.18; 10.14)	ns
Cold rating hand dorsum	13	2.12 ± 1.45 [1.25; 3)	19	3.41 ± 1.44 (2.71; 4.10)	*p* = 0.009
N2 foot (ms)	8	668.13 ± 131.56 (558.14; 778.11)	18	503.11 ± 47.03 (479.72; 526.50)	<0.001
P2 foot (ms)	8	771.63 ± 156.60 (640.71; 902.54)	18	609.72 ± 52.22 (583.76; 635.69)	0.004
Amplitude foot (µV)	11	5.53 ± 4.08 (2.79; 8.27)	18	7.17 ± 2.83 (5.77; 8.58)	ns
Cold rating foot dorsum	13	1.45 ± 2.02 (0.22; 2.27)	19	3.23 ± 1.58 (2.47; 3.99)	*p* = 0.005
CDT foot *Z* value	13	−1.27 ± 1.43 (−2.14; 0.40)	19	0.04 ± 1 (−0.44; 0.52)	0.011

Group comparisons have been performed with the Mann–Whitney *U*-test and *p*-values shown as “significance level.” PNP, patients with polyneuropathy; CDT, cold detection threshold; CEP, cold-evoked potential. The lower and upper 95% confidence limits are presented in brackets below the mean ± standard deviation. Note that there are discrepancies between the patient numbers for latencies and amplitudes. This results from the circumstance that some patients presented clearly abolished potentials, to whom we assigned an amplitude of 0 µV.

**Table 3:** CEP data, QST *z*-values, and questionnaire scores for each patient group after age matching.

	FP		PNP		Significance level (*p*)
	*n*	mean ± SD	*n*	mean ± SD	
N2 foot (ms)	10	499.3 ± 110.8 [420; 578.6]	8	668.1 ± 131.6 [558.1; 778.1]	0.016
P2 foot (ms)	10	596.8 ± 104.4 [522.1; 671.5]	8	771.6 ± 156.6 [640.7; 902.5]	0.012
Amplitude foot (µV)	11	7.6 ± 3.5 [5.2; 9.9]	11	5.5 ± 4.1 [2.8;8.3]	n.s.
PDQ score	13	18.46 ± 7.03 [14.21; 22.71]	13	15.77 ± 8.43 [10.68; 20.86]	n.s.
CDT foot *Z* value	13	−1.7 ± 1.2 [−2.4; −1]	13	−1.3 ± 1.4 [−2.1;0.4]	n.s.
MDT *z*-score	13	−1.3 ± 1.2 [−2; −0.6]	13	−2.8 ± 1.5 [−3.7; −1.9]	0.012
MPT *z*-score	13	1.5 ± 1.1 [0.8; 2.2]	13	−0.2 ± 2.8 [−1.9; 1.6]	0.01
VDT *z*-score	13	−0.5 ± 1.5 [−1.4; 0.4]	13	−3.8 ± 2 [−5; −2.6]	<0.001
HR variability resting		4.4 ± 2.5 [2.9; 5.9]		3.3 ± 1.6 [2.3; 4.2]	n.s.
RMSSD resting		28.8 ± 15.8 [19.2; 38.4]		21.9 ± 10.8 [15.3; 28.4]	n.s.
HR variability breathing		8.9 ± 5.1 [5.8; 11.9]		8.2 ± 7.3 [3.7; 12.6]	n.s.
RMSSD breathing		41.6 ± 20.6 [29.2; 54]		41.2 ± 28.7 [23.8; 58.5]	n.s.
Orthostatic TDM		1.3 ± 0.4 [1; 1.5]		1.2 ± 0.2 [1.1; 1.3]	n.s.

Group comparisons have been performed with the Mann–Whitney *U*-test and *p*-values shown as “significance level.” FD, patients with Fabry disease; PNP, patients with polyneuropathy; PDQ, painDETECT questionnaire; QST, quantitative sensory testing; RMSSD, root mean square of successive square differences; TDM, time-domain measurement; VDT, vibration detection threshold; CDT, cold detection threshold; CEP, cold-evoked potential; HR, heart rate; MDT, mechanical detection threshold; MPT, mechanical pain threshold. The lower and upper 95% confidence limits are presented in brackets below the mean ± standard deviation. Note that there are discrepancies between the patient numbers for latencies and amplitudes. This results from the circumstance that some patients presented clearly abolished potentials, to whom we assigned an amplitude of 0 µV.

#### 3.1.1 Correlation analyses including all patients

As proof of concept, we correlated N2 latency and height, which showed a significant positive correlation, i.e., the taller the subject, the longer the latency (*r* = 0.578; *p* = 0.003). The correlation analysis of both the autonomic measures (i.e., RMSSDb and orthostatic TDM) showed a significant positive correlation (*r* = 0.0626; *p* = 0.000035), confirming their coherent informative value.

**Figure 6:**
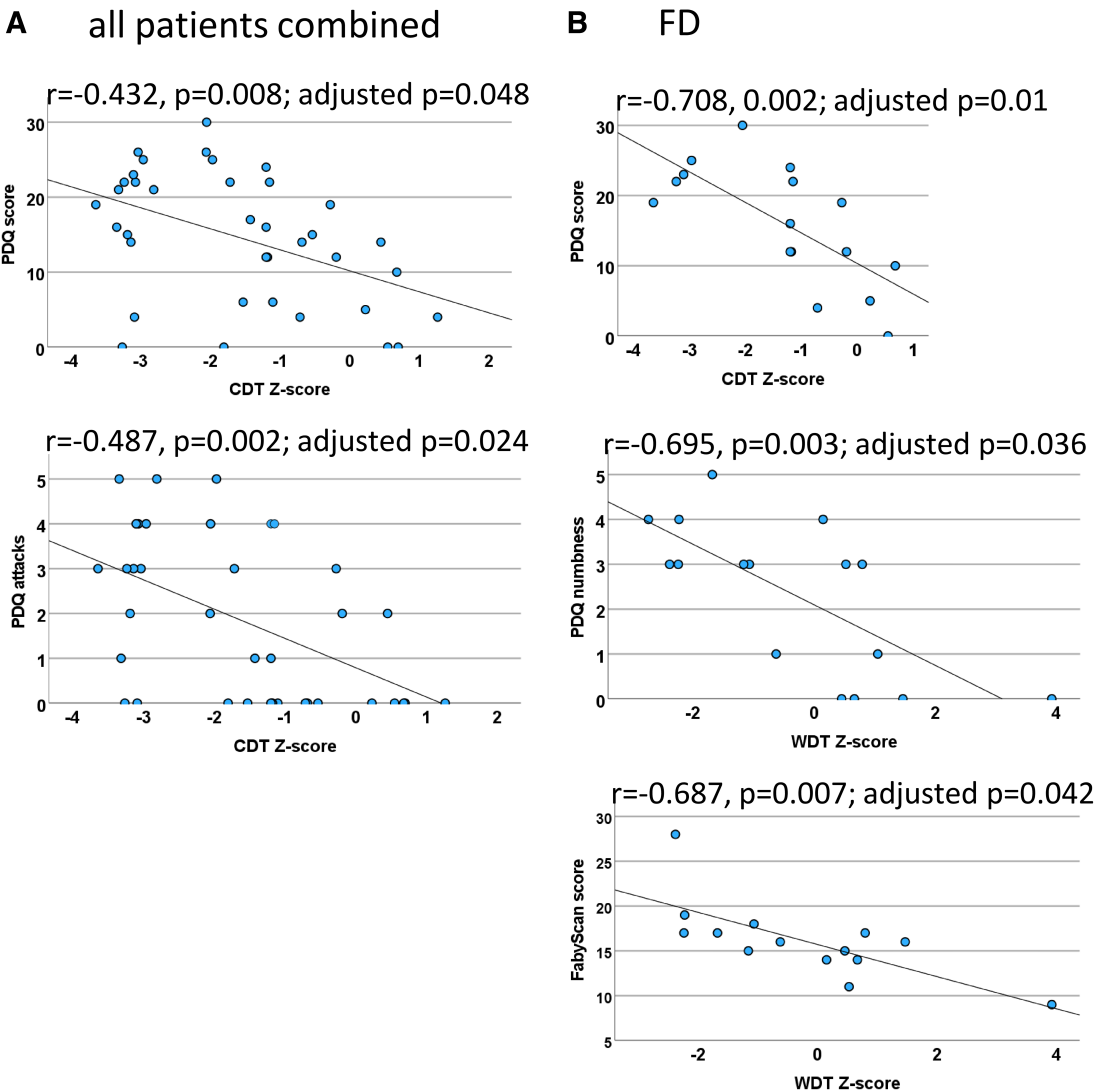
Quantitative sensory testing (QST) correlations. Scatter plots for the significant QST correlations (which withstood correction for multiple testing) within both patient groups combined (**A**) and FD patients (**B**). In the case of the PNP patients, the calculations did not withstand correction for multiple testing. (**A**) The correlation between CDT and the PDQ score indicates that a loss of cold fiber function is associated with a neuropathic pain (NP) component and the sensory symptom of pain attacks. (**B**) Cold fiber function loss is strongly correlated with NP in FD patients. The loss of C-fiber function was associated with higher ratings in the FabryScan and a stronger sensation of numbness. CDT, cold detection threshold; WDT, warm detection threshold; QST, quantitative sensory testing; FD, Fabry disease; PNP, polyneuropathy; PDQ, painDETECT questionnaire.

**Figure 7:**
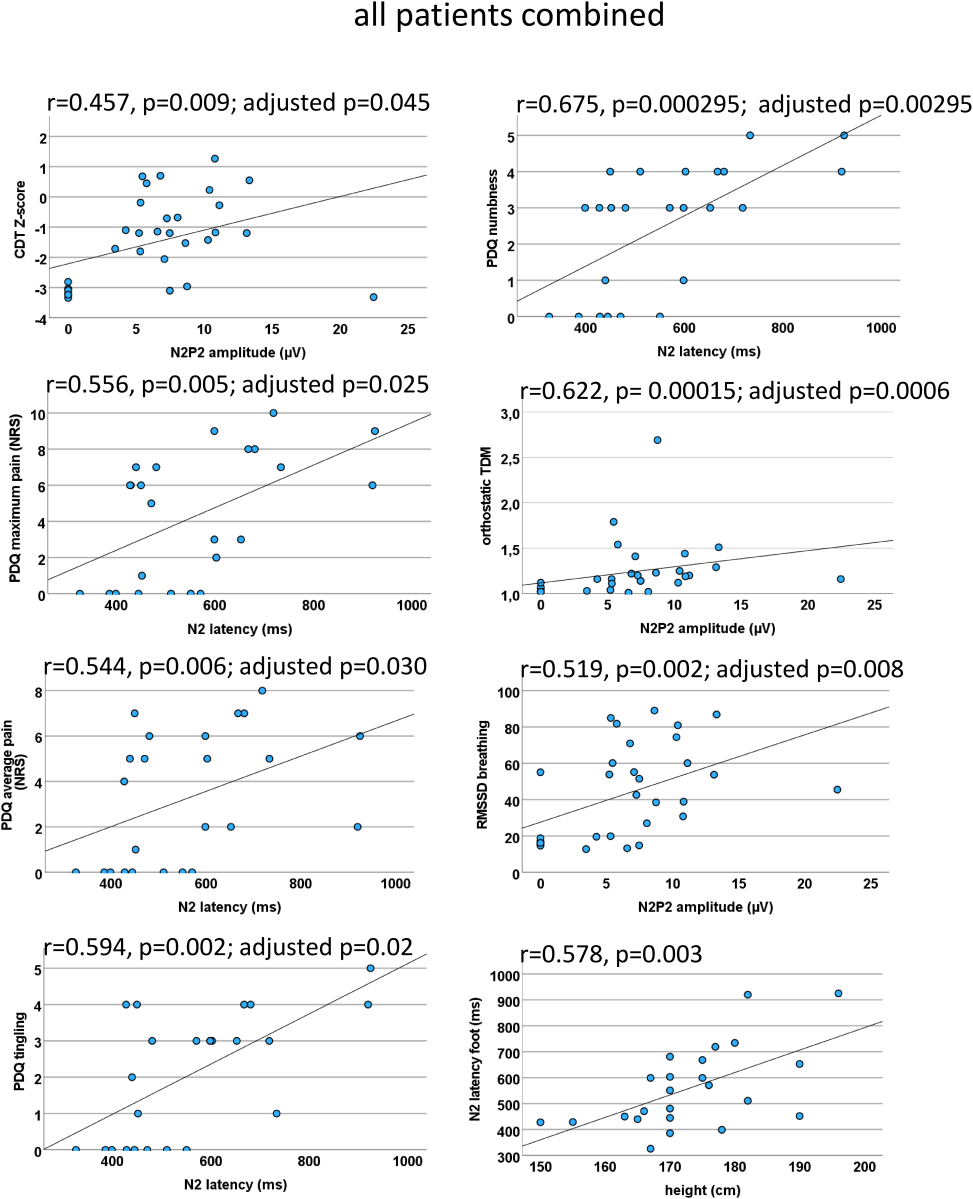
EP correlations within all patients combined. Scatter plots for the significant CEP correlations (which withstood correction for multiple testing) within both patient groups combined. A loss of function indicated by negative CDT *Z*-scores was associated with a CEP amplitude reduction. A prolonged N2 latency was indicative of higher average and maximum pain as well as stronger tingling sensation and numbness. Interestingly, a CEP amplitude decrease was associated with a functional loss of the autonomic nervous system. As a proof of concept, N2 correlated significantly with height (bottom right). CEP, cold-evoked potential; CDT, cold detection threshold; NRS, numeric rating scale; PDQ, painDETECT questionnaire; RMSSD, root mean square of successive square differences; TDM, time-domain measurement.

Figure 6 shows the scatter plots for the significant QST correlations (after correction for multiple testing) and Figure 7 for the CEP correlations within all patients combined.

**QST and pain** *[CDT, warm detection threshold (WDT), mechanical detection threshold (MDT), vibration detection threshold (VDT), mechanical pain threshold (MPT), current pain, max pain, average pain]*

After correction for multiple testing, no significant correlations were found.

**QST and PDQ** (*CDT, WDT, MDT, VDT, MPT, PDQ sum score*)

Of the abovementioned QST parameters, a significant correlation was only found between CDT at the foot and the PDQ score (*r* = −0.432, *p* = 0.008; adjusted *p* = 0.048), indicating that a neuropathic component is associated with a loss of function of cold-mediating fibers.

**QST and painDETECT sensory items** (*CDT, WDT, MDT, VDT, MPT, seven sensory questions*)

After correction for multiple testing, a significant correlation has only been found between CDT and the item “do you have sudden pain attacks” (inverse, *r* = −0.487, *p* = 0.002; adjusted *p* = 0.024).

**CEP and QST items** (*N2 latency, N2P2 amplitude, CDT, VDT, MPT*)

We found a correlation between the CEP amplitude CDT (*r* = 0.457, *p* = 0.009; adjusted *p* = 0.045) and the MPT (*r* = 0.497, *p* = 0.01; adjusted *p* = 0.05), which did not reach valid significance after correction for multiple testing.

**CEP and pain** (*N2 latency, N2P2 amplitude, current pain, max pain, average pain*)

There was a moderate correlation between N2 latency and current pain (*r* = 0.508, *p* = 0.011; adjusted *p* = 0.055 not reaching significance), maximum pain (*r* = 0.556; *p* = 0.005; adjusted *p* = 0.025), and average pain (*r* = 0.544, *p* = 0.006; adjusted *p* = 0.030).

**CEP and painDETECT** (*N2 latency, N2P2 amplitude, seven sensory questions, PDQ sum score*)

When analyzing the seven PDQ questions separately, we found a significant correlation of the N2 latency with the painDETECT question “do you have a tingling sensation” (*r* = 0.594, *p* = 0.002; adjusted *p* = 0.02) and “do you suffer from a sensation of numbness”(*r* = 0.675, *p* = 0.000295; adjusted *p* = 0.00295). After correction for multiple testing, no other significant correlations were found.

**CEP and autonomic measures** (*N2 latency, N2P2 amplitude, RMSSDb, orthostatic TDM*)

The N2P2 amplitude correlated with RMSSD during breathing (*r* = 0.519, *p* = 0.002; adjusted *p* = 0.008) and with the orthostatic TDM (*r* = 0.622, *p* = 0.00015; adjusted *p* = 0.0006), indicating that patients with autonomic dysfunction also present small fiber loss of function.

### 3.2 FD patients

There were no significant differences in CEP and QST data between patients with and without enzyme replacement therapy.

#### 3.2.1 QST results

Abnormal *z* values were detected for CDT in 5/16 (31.3%), cold pain threshold (CPT) in 0/16, WDT in 5/15 (31.3%), heat pain threshold (HPT) in 4/16 (25%), VDT in 4/16 (25%), MDT in 5/16 (31.3%), and finally MPT in 6/16 (37.5%) patients. Compared to our healthy cohort, the MWU indicated a highly significant difference for the CDT (FD −1.29 ± 1.37 vs. controls 0.038 ± 0.99; *p* = 0.003) and MPT (FD 1.51 ± 1.06 vs. controls 0.49 ± 0.94; *p* = 0.006) at the foot.

#### 3.2.2 CEPs and cold ratings

Thirteen data sets (81.25%) of the hand and 14 data sets (87.5%) of the foot complied with our inclusion criteria for the statistical analysis. Out of these datasets, 38.5% of the N2 latencies, 23.1% of the P2 latencies, and 23.1% of the amplitudes of the hand dorsum were abnormal as defined in the experimental procedures. At the foot, 28.6% of the N2 and P2 latencies and 21.4% of the amplitudes were abnormal. In our analysis putting patient EP data into perspective with age-matched controls, the MWU revealed no significant differences. FD patients reported a mean cold rating of 2.6 (±2.27 SD) at the foot and 2.9 (±1.95 SD) at the hand dorsum. Their cold ratings were not significantly different from the controls. See also Table 2 for the descriptive statistics.

#### 3.2.3 Correlation analyses

**Figure 8:**
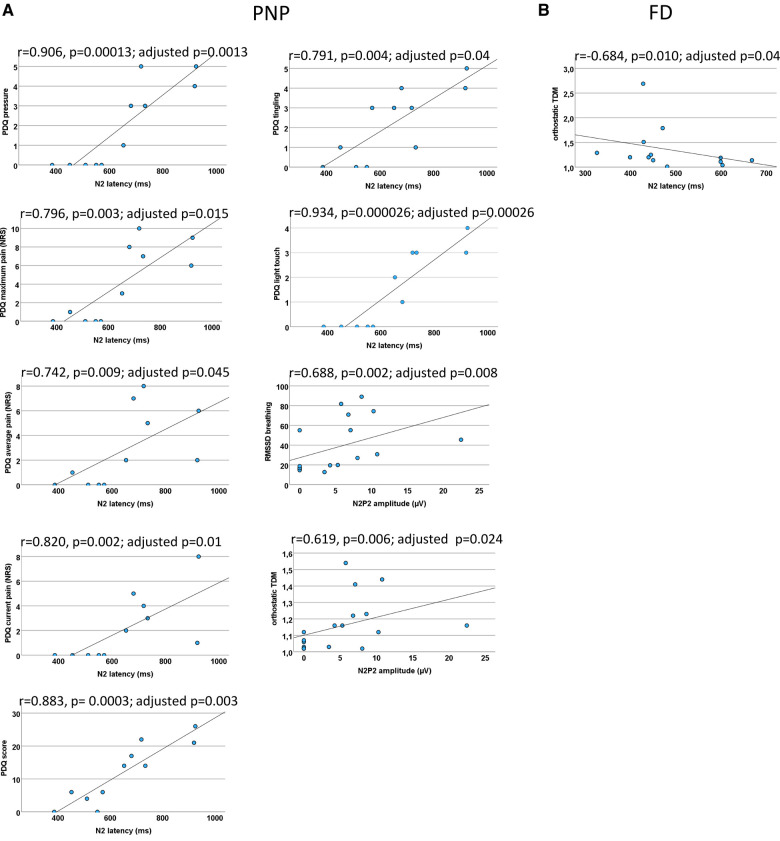
EP correlations for PNP and FD patients each. Scatter plots for the significant CEP correlations (which withstood correction for multiple testing) within PNP patients (**A**) and FD patients (**B**) each. (**A**) A prolonged N2 latency was associated with neuropathic pain (NP) and sensory symptoms (PDQ score and PDQ items). A CEP amplitude reduction was associated with a functional loss of the autonomic nervous system. (**B**) In FD patients, a prolonged N2 latency was associated with a functional loss of the autonomic nervous system. There were no other significant correlations. CEP, cold-evoked potential; FD, Fabry disease; NRS, numeric rating scale; PDQ, painDETECT questionnaire; PNP, polyneuropathy; RMSSD, root mean square of successive square differences; TDM, time-domain measurement.

Figure 6 shows the scatter plots for the significant QST correlations (after correction for multiple testing) and Figure 8 for the CEP correlations within FD patients.

**QST and pain** (*CDT, WDT, MDT, VDT, MPT, current pain, max pain, average pain*)

No significant correlations were found.

**QST and PDQ** (*CDT, WDT, MDT, VDT, MPT, PDQ sum score*)

Within the Fabry group, there was a significant inverse correlation between the CDT at the foot and the PDQ score (*r* = −0.708, 0.002; adjusted *p* = 0.01).

**QST and painDETECT sensory items** (*CDT, WDT, MDT, VDT, MPT, seven sensory questions*)

After correction for multiple testing, a significant correlation has only been found between WDT and the item “do you suffer from a sensation of numbness” (*r* = −0.695, *p* = 0.003; adjusted *p* = 0.036).

**QST and FabryScan** (*FabryScan score, CDT, WDT, MDT, VDT, MPT*)

Within the abovementioned QST parameters, only WDT showed a significant inverse correlation (*r* = −0.687, *p* = 0.007; adjusted *p* = 0.042).

**QST and autonomic measures** (*CDT, WDT, MDT, VDT, MPT, RMSSDb, orthostatic TDM*)

After correction for multiple testing, no significant correlations were found.

**CEP and QST items** (*N2 latency, N2P2 amplitude, CDT, VDT, MPT*)

No significant correlations were found.

**CEP and pain** (*N2 latency, N2P2 amplitude, current pain, max pain, average pain*)

N2 latency correlated with the average pain (*r* = 0.679, *p* = 0.011; adjusted *p* = 0.055), just not reaching significance after correction for multiple testing.

**CEP and painDETECT** (*N2 latency, N2P2 amplitude, seven sensory questions, PDQ score*)

No significant correlations were found.

**CEP and autonomic measures** (*N2 latency, N2P2 amplitude, RMSSDb, orthostatic TDM*)

N2 correlated inversely with the results of the orthostatic TDM (*r* = −0.684, *p* = 0.010; adjusted *p* = 0.04), indicating that FD patients with autonomic dysfunction were associated with abnormal small fiber function.

#### 3.2.4 Subgroup analysis of CEP data

Of our 13 FD patients whose CEP data of the hand were included in the statistical analysis, 5 presented abnormal N2 latencies and 3 abnormal amplitudes. Of the 14 patients whose data of the foot were included, 4 presented abnormal N2 latencies and 3 abnormal amplitudes. Eleven FD patients presented current pain of numeric rating scale (NRS) ≥1, and three FD patients presented no current pain (NRS = 0). The subgroup analyses comparing the patient groups with or without CEP abnormalities at the hand or foot, or with or without current pain, showed no significant differences in terms of the total score of the FabryScan, PDQ score, or the pain items or the CDT.

### 3.3 Patients with PNP

#### 3.3.1 QST results

Mirroring the PNP, abnormal *z*-values were detected for CDT in 11/21 (52.4%), WDT in 5/21 (23.81%), CPT in none, HPT in 3/21 (14.3%), VDT in 18/21 (85.7%), MDT in 14/21 (66.7%), and finally MPT in 6/21 (28.6%) patients. Between age-matched PNP patients and controls, the MWU showed a significant difference in CDT (*p* = 0.011), WDT (*p* = 0.002), CPT (*p* = 0.006), HPT (*p* = 0.016), and finally MDT and VDT (*p* < 0.001 each).

#### 3.3.2 CEPs and cold ratings

Sixteen out of 21 CEP data sets from the hand (76.2%, one exhibiting entirely abolished CEPs) and 18 out of 21 from the foot (85.7%, seven of them presenting entirely abolished CEPs) met our inclusion criteria for statistical analysis. After age matching, 11 data sets (52.4%) remained for analysis of the hand and foot, respectively. For the hand, 54.5% of the N2 and P2 latencies each and 63.6% of the N2P2 amplitudes were abnormal. For the foot, 87.5% of the N2 latencies, 75% of the P2 latencies, and 36.4% of the N2P2 amplitudes were abnormal. The MWU showed a significant difference between PNP patients and controls for the N2 (*p* < 0.001) and P2 (*p* = 0.004) latency. PNP patients reported a mean cold rating of 1.36 (±1.75 SD) at the foot and 2.07 (±1.47 SD) at the hand. In the age-matched comparisons, their cold ratings were significantly different (i.e., lower) from the controls both at the foot (*p* = 0.005) and the hand dorsum (*p* = 0.009). See also Table 2 for the descriptive statistics.

#### 3.3.3 Correlation analyses

Figure 8 shows the scatter plots for the significant CEP correlations (after correction for multiple testing) within PNP patients.

**QST and pain** (*CDT, WDT, MDT, VDT, MPT, current pain, max pain, average pain*)

No significant correlations were found.

**QST and PDQ** (*CDT, WDT, MDT, VDT, MPT, PDQ sum score*)

No correlations were found between QST and the PDQ sum score.

**QST and painDETECT sensory items** (*CDT, WDT, MDT, VDT, MPT, seven sensory questions*)

After correction for multiple testing, no significant correlations were identified.

**QST and autonomic measures** (*CDT, WDT, MDT, VDT, MPT, RMSSDb, orthostatic TDM*)

After correction for multiple testing, no significant correlations were identified.

**CEP and QST items** (*N2 latency, N2P2 amplitude, CDT, VDT, MPT*)

A correlation between the N2P2 amplitude of the foot and CDT was observed but did not withstand correction for multiple testing (*r* = 0.558, *p* = 0.016; adjusted *p* = 0.08). No other significant correlations were found.

**CEP and pain** (*N2 latency, N2P2 amplitude, current pain, max pain, average pain*)

N2 significantly correlated with current pain (*r* = 0.820, *p* = 0.002; adjusted *p* = 0.01), maximum pain (*r* = 0.796, *p* = 0.003; adjusted *p* = 0.015), and average pain (*r* = 0.742, *p* = 0.009; adjusted *p* = 0.045).

**CEP and painDETECT** (*N2 latency, N2P2 amplitude, seven sensory questions, PDQ score*)

The N2 latency correlated significantly with the painDETECT questions “is light touch painful” (*r* = 0.934, *p* = 0.000026; adjusted *p* = 0.00026), “does slight pressure trigger pain” (*r* = 0.906, *p* = 0.00013; adjusted *p* = 0.0013), and “do you have a tingling sensation” (*r* = 0.791, *p* = 0.004, adjusted *p* = 0.04) and finally with the painDETECT sum score (*r* = 0.883, *p* = 0.0003; adjusted *p* = 0.003). All other results did not withstand correction for multiple testing.

**CEP and autonomic measures** (*N2 latency, N2P2 amplitude, RMSSDb, orthostatic TDM*)

The N2P2 amplitude correlated significantly with RMSSD during forced breathing (*r* = 0.688, *p* = 0.002; adjusted *p* = 0.008) and orthostatic TDM (*r* = 0.619, *p* = 0.006; adjusted *p* = 0.024).

#### 3.3.4 Subgroup analysis of CEP data

The comparison of painful (*n* = 16) vs. painless (*n* = 5) PNP patients showed no significant differences in latencies or amplitudes. When compared to age-matched controls (*n* = 19), patients with painful PNP (*n* = 8) exhibited significantly longer N2 and P2 latencies (*p* < .001 each) at the foot.

### 3.4 Comparison of patient groups

As shown in Table 3 and Figure 5, PNP patients exhibited significantly longer CEP latencies (indicating a loss of small fiber function) and a more pronounced loss of large fiber function (MDT, VDT) compared to the FD group. Interestingly, MPT was in the gain range of the QST *Z*-scores in FD patients and the loss range in PNP patients.

## 4 Discussion

The main goals of this study were to test the feasibility of CEPs in two patient groups and to identify clinical or sensory features associated with CEP abnormalities. By presenting data of patients with FD, a typical small fiber disorder, and patients with PNP, a typical mixed fiber condition, we hereby contribute new insights to the controversy of the clinical usefulness of CEPs. We found (1) a consistent informative quality in neurophysiological (i.e., CEPs) and psychophysical (i.e., QST) diagnostic measures and (2) an association of CEP data with self-reported sensory symptoms and a NP component, as reflected by the PDQ and finally (3) an association of CEP data (but not QST) with autonomic measures in both patient groups. The results are encouraging regarding the use of CEPs as an electrophysiological tool to detect NP in clinical practice, which will be discussed further below.

As Figure 8 shows, these associations varied among our patient groups. Although the functional loss of cold-mediating fibers is reported as a typical feature of FD (18, 20, 21), this loss of cold fiber function was more pronounced in our PNP group, suggesting an overall disease progression in this patient cohort with dysfunction of large and small fibers. The functional loss within the cold-mediating small fibers in PNP patients was reflected by significantly prolonged CEP latencies as well as in the QST profile. For instance, 52% of the PNP patients exhibited abnormal CDT and 86% abnormal VDT, compared to only 31% of FD patients with abnormal CDT and 25% with abnormal VDT. In summary, the QST results for the PNP group pointed to a somatosensory nervous system with more severe dysfunction, especially within the large fiber range—a finding consistent with the expected characteristic of PNP as noted in previous studies (4, 7).

Through the correlation analyses and group comparisons with CEP and QST data, patient-reported outcome measures (PROMs), and autonomous measures, several interesting observations were made as follows:

1) CEP amplitudes showed a significant correlation with the CDT when including all patients in the analysis, i.e., a reduced N2P2 amplitude was indicative of a loss of function as detected by the CDT within the QST protocol. This finding illustrates CEPs' capability to assess small fiber neuropathy through an impaired function of cool-sensitive Aδ fibers. These results are consistent with previous studies using noxious stimuli to examine NP in patients with FD [i.e., pain-related evoked potentials (PREPs) and LEPs (55, 56)]. In our cohort, when analyzing FD patients alone, there were no significant correlations between the CEP data and the QST items. The absence of correlation may be explained by a more intact sensory nervous system in FD patients compared to the PNP patients, or it could suggest that sensory function in FD patients varies more dynamically depending on pain exacerbations.

CEP latencies correlated significantly with current, maximum, and average pain, when including all patients in the analysis or when analyzing PNP patients alone. Even in the smaller FD group, there was a significant correlation with the average pain (notwithstanding correction for multiple testing). This finding is particularly noteworthy, as the functional loss of cold-mediating fibers appears to be indicative of the development of (chronic) pain (57–59). However, due to the small cohort, it is not possible to conclude mechanistic conclusions about ongoing spontaneous NP from our data and further studies with larger patient cohorts are necessary. Nonetheless, the following considerations can be made: CEPs allow for the examination of specialized, cool-sensitive Aδ- and C-fibers (60–62) and their central pathways (26, 28, 30, 31). While the conduction velocity offers insights into the state of the myelin sheaths, changes in the amplitudes can be due to axonal dysfunction or, as shown for nociceptive pathways (63), sensitization processes. Our findings illustrate how small fiber damage, reported by PROMs and assessed through psychophysiological methods, can indeed be mirrored by *non-noxious*, quantifiable electrophysiological parameters as shown here by CEP latencies and CEP amplitudes. A prolonged latency could result from distal loss of peripheral thermosensory input or perhaps the desynchronization of ascending information (64). In this case, the positive correlation of CEP latencies of the PNP group with the reported pain intensity suggests that the same mechanisms leading to an impairment of specialized, cool-sensitive Aδ-fibers will also affect nociceptors. Furthermore, our CEP data seem to support the mechanistic approach of central disinhibition pain that is thoroughly discussed in the review by Forstenpointner et al. (65) and comprises the disbalance between the lateral and the medial pain pathway, two phylogenetically different pain processing systems. An impaired Aδ fiber function may have an “unmasking” effect on the nociceptive C-fibers in our PNP group, notably where large fibers exhibited pronounced dysfunction.

2) CEP latencies showed a certain association with somatosensory signs and symptoms. The PDQ contains two questions reflecting dynamic mechanical allodynia [proposed as an important clinical marker for central sensitization (66)]: “is light touch painful (…)?” and “does slight pressure trigger pain (…)?”. We found a highly significant correlation between these items and N2 latency within the PNP cohort (see Figure 8). These findings may encourage further studies to explore a possible link between abnormal CEP latencies and the presence of central sensitization in PNP patients.

In this patient group, the CEP latency also correlated strongly with the item “do you have a tingling sensation” and the painDETECT sum score, suggesting an association of prolonged CEP latencies with a higher likelihood of the presence of an NP component [*note: the higher the PDQ score, the higher the likelihood of NP* (52)] and the occurrence of pain in this group. This is further supported by a subgroup analysis, showing that PNP patients with current pain presented significantly longer N2 latencies than those in age-matched controls, while the CEP data of painless PNP patients did not differ significantly from age-matched controls. These results, however, must be interpreted with caution due to the small number of painless PNP patients (*n* = 3). Nonetheless, our results are in line with previous reports on altered N2 latencies in NP conditions assessed through other evoked potentials (67, 68). In a previous study, we demonstrated how the N2 latency of LEPs in radiculopathy patients correlated with pain intensity and clinical severity in the affected dermatome (69). The FD group on the other side showed no correlation of CEP data with the items of the PDQ assessing sensory signs and symptoms, emphasizing the value of QST and how the examined methods are not interchangeable.

3) Abnormal CEPs indicated abnormal autonomic small fiber function, possibly leading to direct diagnostic consequences for further assessment and possible therapeutic implications. The analysis of the entire patient cohort and the PNP group alone revealed a significant correlation between the N2P2 amplitude of the foot and both the RMSSDb and orthostatic TDM. Similarly, the analysis of the FD group revealed an inverse correlation between the N2 latency and the orthostatic TDM. These findings consistently suggest that the electrophysiological integrity of intact cold-mediating fibers is linked to the functional integrity of the autonomic nervous system. In other words, a low CEP amplitude indicates a loss of function of the cold-mediating fibers, and lower RMSSD and TDM scores indicate abnormal parasympathetic/autonomic small fiber function, possibly with a direct diagnostic consequence of further assessment thereof and potential therapeutic implications. This correlation aligns with previous reports on the simultaneous involvement of somatic and autonomic small fibers in autonomic neuropathies (70). Although Thaisetthawatkul et al. (71) reported that somatic and autonomic small fibers require independent and complementary measures, their conclusion relied upon data acquired by QST. In this study, we have now been able to show that CEPs might provide information not only about cold-mediating fibers and their central pathways but, indirectly, also about the status of autonomic small fibers. In their consensus statement on the electrodiagnostic assessment of the autonomic nervous system, Cheshire et al. (72) stated that the “evaluation of disorders of the autonomic nervous system is both an art and a science, calling upon the physician's most astute clinical skills as well as knowledge of autonomic neurology and physiology.” Our findings propose that abnormal CEPs may signal abnormal autonomic function, warranting further autonomic diagnostic tests upon detecting abnormal CEP parameters.
4) Our correlation analyses with the QST data showed differing results for FD and PNP patients. In patients with FD, the significant inverse correlation between WDT and the FabryScan suggested an association between loss of function of warm-mediating small fibers and an increased likelihood of screening positive for FD. Similarly, we observed an association between a functional loss of cold-mediating small fibers and an increased probability of having a NP component (see also Figures 1, 6). Notably, these associations did not emerge among PNP patients. The self-reported pain intensities (current, maximum, and average pain) in neither the FD nor PNP patients were linked with QST parameters except for WDT and the item “do you suffer from a sensation of numbness” in FD patients. These findings seem to support a potential beneficiary capacity of CEPs, mirroring clinical indicators of chronic pain and providing insight into the cold-mediating pathways.

Interestingly, the MPT significantly varied between the FD and PNP groups, a metric associated with central sensitization (73, 74). The FD group's MPT fell within the *Z*-score's gain range, whereas the PNP group's MPT was in the loss range, indicating a pronounced sensory loss (and a progressed chronic state of the disease) in the PNP group, while FD patients showed stronger signs of central sensitization. A similar observation was observed previously in patients with painful radiculopathy, where signs of central sensitization were present in the early stages of the disease, which then were replaced by functional loss of the pain-mediating nerve fibers with disease progression (69).

### 4.1 Study limitations

Despite some patients not meeting EEG quality criteria for statistical analysis, we successfully conducted CEP recording and evaluation in over 80% of participants (Table 1). To comprehensively address the posed questions, further investigation in larger patient cohorts is essential. In line with previous reports on healthy individuals, our present study shows how the examination of CEPs has its technical limitations, i.e., CEPs were not recordable in all our patients due to various reasons (see above). Particularly, examining the distal lower extremities in elderly subjects remains a challenge (31). While improvements in CEP recording could be achieved with advanced thermal stimulators (featuring steep cooling ramps and low target temperatures (35, 75), for now, only the Medoc thermal stimulator possesses a CE certificate as a diagnostic tool on patients in clinical routine, facilitating their application in both research and clinical diagnostics.

### 4.2 Why is it worthwhile to pursue research efforts toward the clinical applicability of CEPs?

As described above, early FD detection is crucial to prevent disease progression, emphasizing the need for diagnostic precision in identifying early-phase small fiber dysfunction. This is a notable challenge in clinical practice. Beyond QST and CEPs, various research groups focused on microneurography to gain a readout of specific sub-types of sensory nerve fibers (76–78). This method also allowed the description of a subset of cold-mediating C-fibers (79–81). Due to its time-consuming nature, its availability is only limited and unfit for routine usage in patients. Thus, microneurography remains a tool of few, specialized research centers. Currently, the gold standard for the electrophysiological assessment of small fibers is LEPs (3, 32). Both LEPs and contact-heat evoked potentials (CHEPs) (69, 82–85), allow an examination of the thermo-nociceptive nervous system by visualizing the pain-related brain potentials within the EEG (86). The quantifiable and reliable feature assigns these tools a potentially decisive stance for the classification of a given pain syndrome as “definitely neuropathic” (25, 87). Unfortunately, though, LEPs also failed to reach a broad clinical utilization partly due to potential skin damage and necessary safety precautions. This is where CEPs might step in: The advantages of CEP assessment as a non-invasive method to measure small fibers and their central pathways are comparable to those of LEPs, but with the upside of a pain-free examination. A study on the conduction velocity of the cold spinal pathway even suggested that CEPs may represent an alternative to LEPs (75).

Although we are not quite there yet, the appeal of electrophysiological spinothalamic tract examinations without painful stimuli is particularly attractive for sensitive or hyperalgesic skin areas in clinical settings, though further advancements are needed to integrate CEPs into routine electrophysiological diagnostics, necessitating enhanced CEP paradigms across diverse NP conditions and robust normative data collection. Notably, our results point to the capacity of CEPs to indirectly assess the status of autonomic small fibers. This observation is worth examining in further studies.

## 5 Conclusions

CEPs were successfully obtained from patients with NP and correlated with both QST results and PROMs. As expected, the application of CEPs and QST revealed that the somatosensory system of the PNP group was more severely affected by functional loss compared to the FD group. Moreover, abnormal CEPs, unlike QST, were associated with dysfunctional autonomic nervous system function in both FD and PNP patients. Thus, abnormal CEPs may indicate neuropathic and/or chronic pain conditions and could prompt further diagnostic actions (e.g., autonomic diagnostics), although additional studies are necessary for confirmation. It is worthwhile to further improve CEP paradigms to make CEP accessible to all patients. This may be achieved by modern cold stimulators with steep temperature ramps, so far lacking a CE certificate for a diagnostic and clinical purpose. In summary, CEPs hold significant potential as a diagnostic adjunct for NP.

## Data Availability

The raw data supporting the conclusions of this article will be made available by the authors upon reasonable request.
