# Supplementary material for: Cold-evoked potentials in Fabry disease and polyneuropathy
Source: Front Pain Res (Lausanne). 2024 May 15;5:1352711. doi: 10.3389/fpain.2024.1352711 (PMC11133603; doi:10.3389/fpain.2024.1352711)
Supplement: Supplementary file 1 [file Datasheet1.docx]

# Supplementary Material

## Inclusion and exclusion criteria

General exclusion criteria

- not able to give informed consent
- insufficient language skills
- alcohol- or drug abuse
- pregnancy or lactation
- participation in drug trial <30 days before inclusion

Inclusion criteria for polyneuropathy patients (England et. al.)

- presence of neuropathic signs and/or symptoms
- abnormal electrophysiology or *small fiber testing*

Fabry patients

- mutation in the alpha-Gal-gene
- clinical evaluation in a Fabry center

Exclusion criteria for healthy controls (according to Gierthmühlen et al. 2015, *PMID 26075963*)

- current pain or relevant pain in the last 14 days
- relevant strong pain in the past 3 months, including radiculopathy, headache, local back pain
- analgesic intake on >10 days in the last month
- any analgesic intake in the last 14 days
- intake of triptans in the last 24 months
- migraine with >1 attack in the last 24 months
- history of chronic pain
- frequent work absence due to pain
- psychological or psychiatric treatment for a longer period in the last 5 years
- smoking >39 cigarettes per day
- regular intake of medication, especially psychostimulants (contraceptives, *blood pressure and well-controlled thyroid medication was tolerated*) or St. John´s wort or past intake in the last 7 days
- intake of illegal drugs, including cannabis in the past 4 weeks
- alcohol consumption in the past 48 hours
- energy drink consumption in the past 48 hours
- intake of any drugs with psycho-/neuropharmacological effects in the last 3 months
- severe disorders of the nervous system, the respiratory system, uncontrolled disorders of the circulatory system, gastrointestinal disorders, disorders of liver, gall bladder or pancreas, kidneys or the urogenital system, metabolic disorders, musculoskeletal disorders, hepatitis, HIV
- diagnosed depression, anxiety, panic attacks, eating disorders, chronic fatigue or exhaustion, addiction or dependence, psychosis
- skin diseases including the testing area
- polyneuropathy
- jet lag, irregular working hours or sleep restriction in the last 2 days
- changes in physical exercise 1 week prior to the study
- absent or abnormally diminished Achilles´ tendon reflex
- vibration detection threshold <5/8 <60 years or <4/8 >60 years at the medial malleolus

## Supplementary Tables

Overview of abnormal EEG and QST results in FD patients (**A**) or the age matched PNP patient cohort (**B**) whose EEG data complied with our inclusion criteria at the foot (“abnormal” as defined above and indicated through “x”).

**A**

| **FD patient** | **pain** | **PDQ: NP probable** | **N2- latency** | **N2P2-amplitude** | **CDT** | **MPT** | **WDT** | **HPT** |
| --- | --- | --- | --- | --- | --- | --- | --- | --- |
| Patient 1 | x |  | x | x |  |  | x |  |
| Patient 2 | x | x |  |  |  | x |  |  |
| Patient 4 | x |  | x |  |  | x |  | x |
| Patient 5 | x |  |  |  |  |  | x |  |
| Patient 6 | x | x | x |  | x |  | x | x |
| Patient 7 | x | x |  | x | x |  | x |  |
| Patient 9 | x | x |  |  |  | x |  | x |
| Patient 10 |  |  |  |  |  | x |  |  |
| Patient 11 |  |  |  |  |  |  |  |  |
| Patient 12 |  |  |  |  |  | x |  |  |
| Patient 13 |  |  |  | x |  |  |  |  |
| Patient 14 | x | x |  |  | x | x | x |  |
| Patient 15 | x | x |  |  |  |  |  | x |
| Patient 16 | x |  | x | x |  |  |  |  |

*(Pain: current pain ≥ 1; PDQ: painDETECT questionnaire score above 18; N2-latency: above the upper limit of a 95% confidence interval of age-matched healthy controls’ artefact-free average; N2P2-amplitude: below the lower limit of a 95% confidence interval of age-matched controls’ artefact-free average; CDT/MPT/WDT/HPT: z-value outside the limits of a 95% confidence interval of healthy controls of the DFNS database)*

**B**

| **PNP patient** | **pain** | **PDQ: NP probable** | **N2- latency** | **N2P2-**  **amplitude** | **CDT** | **MPT** | **WDT** | **HPT** |
| --- | --- | --- | --- | --- | --- | --- | --- | --- |
| patient 2 | x |  | x |  |  | x | x |  |
| patient 3 |  |  | x |  |  |  | x |  |
| patient 5 | x |  |  | x | x |  |  |  |
| patient 6 | x | x | x |  | x |  |  |  |
| patient 8 | x |  | x |  |  |  |  |  |
| patient 12 | x | x |  | x | x |  |  |  |
| Patient 16 | x | x |  | x | x | x |  |  |
| Patient 18 | x |  | x |  |  |  |  |  |
| Patient 20 | x | x | x | x |  |  |  |  |
| Patient 21 |  |  |  |  |  |  |  |  |
| Patient 23 |  |  | x |  |  | x |  |  |

*(Pain: current pain ≥ 1; PDQ: painDETECT questionnaire score above 18; N2-latency: above the upper limit of a 95% confidence interval of age-matched healthy controls’ artefact-free average; N2P2-amplitude: below the lower limit of a 95% confidence interval of age-matched controls’ artefact-free average; CDT/MPT/WDT/HPT: z-value outside the limits of a 95% confidence interval of healthy controls of the DFNS database)*

# TSL

*TSL was successfully evaluated, and abnormalities were frequent in our PNP group. There was a significant correlation of the TSL with CEP amplitude (r=0.619; p=0.008, after correction for multiple testing p=0.048) but not with the N2 latency, current, maximum or average pain. TSL abnormalities were discussed to be sensitive markers or even be early predictors of small fiber loss (PMID: 29036361), which further supports our finding that PNP group showed a pronounced small fiber affection.*

*Note:*

*TSL was evaluated in both our patient groups during routine QST testing protocol. Unfortunately, in the FD group, only 4 patients complied with this part of the protocol which is why TSL has not been included in the statistical analysis and considerations of the main manuscript.*
